# Microbial community structure and diversity in fish-flower (mint) symbiosis

**DOI:** 10.1186/s13568-023-01549-4

**Published:** 2023-05-11

**Authors:** Jianglong Wang, Yufen Xie, Guangdi Zhang, Lin Pan

**Affiliations:** School of Food & Wine, Ningxia University, Yinchuan, China

**Keywords:** Mint (*Mentha spicala* L*.*), Carp (*Cyprinus carpio*), Crucian carp (*Carassius auratus*), Grass carp (*Ctenopharyngodon idella*), Microorganism, Aquaponics

## Abstract

The fish-flower symbiosis model is an eco-friendly sustainable farming technology combining plants, fish and microorganisms in a recirculating aquaculture system. However, there are few studies on the structure and diversity of microbial communities in fish intestines, culture water and plant roots during fish-flower symbiosis. Here, we cultured carp (*Cyprinus carpio*), crucian carp (*Carassius auratus*) and grass carp (*Ctenopharyngodon idella*) with mint (*Mentha spicala* L.) and extracted total genomic DNA from intestinal microorganisms, culture-water microorganisms and root microorganisms for each fish species for high-throughput sequencing of 16S rRNA genes. Analysis of microbial community structure and diversity revealed changes in abundance of microbial genera in the intestines and culture water of each fish species, including changes in the dominant taxa. *Pirellula, Truepera, Aquincola, Cetobacterium* and *Luteolibacter* were widespread in the fish intestine, culture water and mint root system. This study revealed the effects of mint feeding on the structure and diversity of microbial communities of fish, water bodies and the mint root system during fish-flower symbiosis, providing a theoretical reference for the promotion and application of fish-flower (mint) symbiosis technology and healthy fish culture technology.

## Introduction

Increased scale and intensification of aquaculture has resulted in heavy use of traditional chemicals and antibiotics. However, this has several drawbacks, including an increase in chemical drug resistance of pathogenic bacteria and the entry of antibiotic residues into the human food chain, which is of public health and environmental concern (Hu et al. 2022; Wei et al. 2022). Therefore, the search for alternative agents with few side effects and minimal environmental pollution is a hot topic of current research. Chinese herbal medicine has received widespread attention because of its low cost, natural ingredients, no drug resistance and no drug residues (Li et al. 2022). Mint (*Mentha spicala* L.) belongs to the genus *Mentha* in the order Labiatae, family Labiatae. The dried aboveground parts can be used medicinally. Mint is distributed throughout China, with artificial cultivation mainly concentrated in Jiangsu and Anhui, the traditional production areas (Dong et al. 2015). There are two main groups of mint cultivars, those rich in menthol and those rich in carvone (Monadi et al. 2021). Mint has been used as a feed additive for *Oncorhynchus mykiss* (Sonmez et al. 2015). In addition, Shete et al. (2016) employed the genus *Mentha* in aquaculture by adjusting feeding regimes and bait composition to improve the digestive enzyme activity and immunity of fish. The fish-flower symbiosis model is a safe and healthy, green model that combines aquaculture technology and plant cultivation technology in one. It can realize the harmonious symbiosis of “fish fertilizing water-flower purifying water-water nourishing fish” and has obvious ecological and environmental advantages over other production methods (Greenfeld et al. 2022). Fish intestines, like those of other animals, contain a complex ecosystem of microorganisms (Wang et al. 2018). Fish maintain a balanced core intestinal flora through beneficial microbial interactions and strain variation, regulating gene expression associated with epithelial cell proliferation and innate immunity (Kokou et al. 2019; Liu et al. 2021a, b). This study aimed to clarify the composition of fish intestinal microflora, rhizosphere microorganisms and water column microorganisms under fish-flower (mint) symbiosis.

We employed three common fish species in aquaculture, namely, carp (*Cyprinus carpio*), crucian carp (*Carassius auratus*) and grass carp (*Ctenopharyngodon idella*), in this experiment, and examined the microfloral structures of fish intestinal microorganisms, culture-water microorganisms and root microflora associated with each fish species under the fish-flower (mint) symbiosis model. Our results provide a theoretical basis for the promotion of healthy fish culture technology from a microbiological perspective.

## Materials and methods

### Materials

The carp (*Cyprinus carpio*), crucian carp (*Carassius auratus*), grass carp (*Ctenopharyngodon idella*) and mint (*Mentha spicala* L.) used were all from the breeding base of Ningxia Yinchuan Kehai Biotechnology Co. (106.36222E, 38.62176N). Healthy carp, crucian carp and grass carp with normal appearance, healthy body condition and weight of 50 ± 2 g were selected. The fish (120 each species) were randomly divided into control and experimental groups and cultured in 300-L round polyethylene culture drums. In the experimental group, ecological pontoons were laid on the water surface of the culture barrel in which mint plants of the same age were cultivated. Each bucket was stocked with 30 fish of the same species, with three replications for each type of fish. In the control group, 10 fish of each of the three species were placed in each bucket in a mixed culture, with three replications. All culture water was collected from the same pond. During the breeding period, the control group was fed the same brand and batch of commercial feed, and the experimental group was fed freshly cut mint stems and leaves. Daily feeding occurred at 09:00 and 17:00, with a daily baiting rate of 3–5% of fish weight. The amount of feeding was adjusted weekly according to the growth of the fish. Samples were taken after 60 days of culture, and feeding was stopped 24 h before sampling. The water was not changed during the test period; water was replenished and supplied with uninterrupted aeration and oxygenation. Water quality was tested every 15 days before feeding. Phosphate, ammonia nitrogen and nitrite nitrogen were measured according to Chinese national environmental protection standards. pH, temperature and dissolved oxygen content (DO) were measured in situ using a water quality instrument (Pocket Pro+ and HQ40; Hach, Loveland, CO, USA).

## Methods

### Sample collection

Three healthy and similarly sized experimental fish were randomly selected from three replicates in each group. The intestinal contents were dissected, mixed and divided, then quickly snap-frozen in liquid nitrogen and stored in a − 80 °C freezer. Water samples were taken from upper, middle and lower positions of the breeding barrel using sterile Plexiglas water collectors, mixed thoroughly, and insoluble particulate impurities were filtered out using a 2–5 μm pore size filter membrane. Samples were collected after vacuum filtration and enrichment through a 0.22 μm filter membrane and stored at − 80 ℃. Plant root samples were collected using sterile forceps, rinsed with culture water to remove root surface sediment, washed with sterile water by shaking, packed into sterile microcentrifuge tubes, and stored at − 80 ℃ for backup. Thirteen samples of microorganisms were analyzed from the intestines (control E1, E2, E3, experimental A14 [carp], H15 [crucian carp], D12 [grass carp]), the water column (control E5, experimental A9 [carp], H1 [crucian carp], D1 [grass carp]) and the mint root system (A12 [carp], H14 [crucian carp], D9 [grass carp]) using high-throughput sequencing.

### DNA extraction and high-throughput sequencing

DNA extraction and sequencing of the samples were performed in collaboration with Biomarker Technologies. Genomic DNA from the samples was examined for quality using 1% (w/v) agarose gel electrophoresis. Quality control analysis and Agilent 2100 assay (Agilent, USA) were performed on total DNA. Specific primers containing barcode 27 F/1492 R were designed against the V3–V4 region of the bacterial 16S rRNA gene. Sample PCR amplification products were homogenized and mixed in equal volumes, and the final products were sequenced on a Sequel sequencer (Biomarker Technologies, Beijing, CHN). PCR amplification conditions were as follows: 27 cycles of pre-denaturation at 95 ℃ for 30 s, annealing at 50 ℃ for 30 s, extension at 72 ℃ for 60 s; extension at 72 ℃ for 70 min (PCR instrument: ABI GeneAmp 9700 model; ABI, USA).

### Statistical and bioinformatics analysis of data

Raw 16S rRNA gene sequences obtained in this study have been submitted to the NCBI Sequence Read Archive database (https://submit.ncbi.nlm.nih.gov/subs/sra/) under the accession number PRJNA832050. Usearch version 11.0.667 (http://www.drive5.com/usearch/) was used to classify sequences and identify multiple operational taxonomic units (OTUs) based on similarity. The RDPclassifier (Zhang et al. 2021a, b, c) Bayesian algorithm was used to analyze the reads of representative OTU sequences sharing 97% similarity. Community composition of the samples was measured at each taxonomic level, and sample sequences were flattened according to the minimum number of sample sequences to obtain normalized data. OTUs with similarity less than 97% were selected to obtain expected dilution curves. Diversity analysis was performed using software MOTHUR 1.34.4 (Jiang et al. 2022) and SPSS 18.0 (IBM, Armonk, NY, USA). Related alpha diversity indices (Chao1, Shannon, Ace) and beta diversity were analyzed using QIIME and UniFrac distance matrix software (Chen et al. 2014). Intestinal microorganisms, changes in microbial abundance in culture water and the structure of the mint root microflora were compared for the three species of fish at the genus level. Differences in the composition and variation characteristics of the microflora were compared using principal component analysis. Te independent sample t-test (SPSS 18.0 [IBM, Armonk, NY, USA]) was used to detect significant differences of water quality parameters. Te correlation between bacteria and test treatments was evaluated using Spearman correlation heatmap analysis (R pheatmap package).

## Results

### Water quality

Compared with the control group, feeding mint in the fish-flower (mint) symbiosis model had different degrees of effect on culture-water quality. Temperatures showed no significant difference between the experimental group and the control group. Ammonia-N, nitrite-N and pH were all reduced in the experimental group compared with the control group, but the differences in levels were not significant. Three water quality parameters, DO, phosphate and alkalinity, showed significant changes in the experimental group compared with the control group. DO was significantly higher in the carp and crucian carp experimental groups compared with controls. There was a significant decrease in alkalinity in crucian carp culture water, which was the opposite of the slight increase in alkalinity in the other two fish groups. Differences in water quality between the experimental and control groups were mainly in phosphate level. Phosphate levels in the culture water of the three fish species fed with mint were significantly higher than those in the control group, especially the phosphate in the crucian carp group (Table 1).

**Table 1:** Quality assessment of water for the control and test groups

Sample	DO (mg L^−1^)	Temperature (℃)	Ammonia-N (mg L^−1^)	Nitrite-N (mg L^−1^)	pH	Phosphate (mg L^−1^)	Alkalinity (mg L^−1^)
Control group	5.58 ± 0.72	25.53 ± 1.39	0.49 ± 0.41	0.13 ± 0.10	8.60 ± 0.22	0.08 ± 0.03	261 ± 63.04
Carp	7.52 ± 0.77*	26.54 ± 0.66	0.22 ± 0.08	0.06 ± 0.07	8.29 ± 0.20	0.36 ± 0.15*	275 ± 27.25
Crucian carp	8.08 ± 0.55*	26.76 ± 0.63	0.46 ± 0.13	0.09 ± 0.04	8.06 ± 0.46	0.59 ± 0.09*	181 ± 45.22*
Grass carp	5.59 ± 0.68	26.08 ± 0.83	0.30 ± 0.21	0.07 ± 0.07	8.19 ± 0.59	0.37 ± 0.06*	277 ± 49.91

^*^Significant difference compared with the control group; p < 0.05

### Sequencing data analysis

**Fig. 1:**
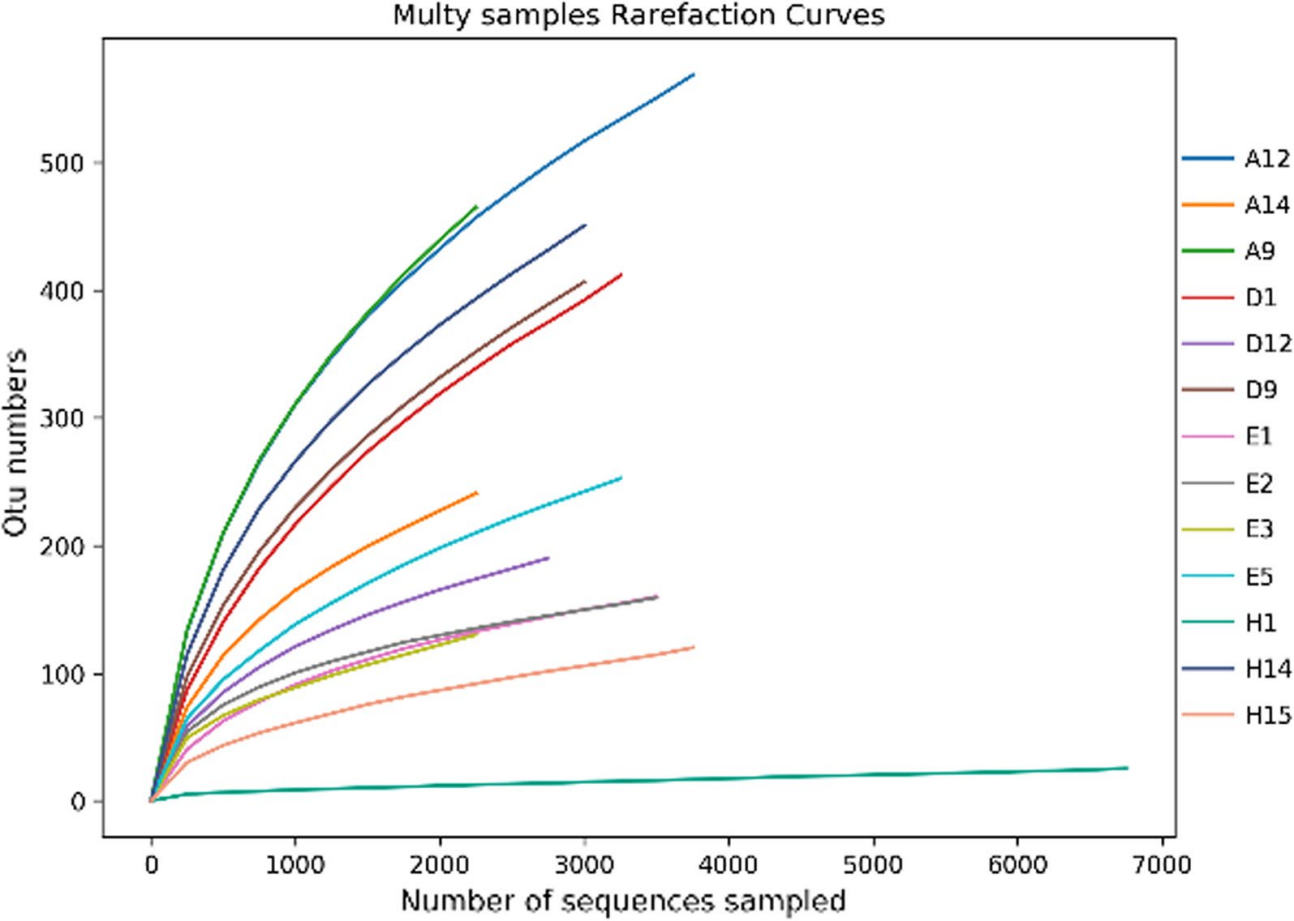
Dilution Curve of Sample

After quality-control filtering and removal of chimeras, a total of 56,211 effective tags and 44,952 base sequences (reads) were obtained, with an average length of 1441.08 bp; bacterial sequence lengths were highly concentrated in the range of 1440 to 1470 bp. Effective tags were clustered at 97% similarity level, and 3600 OTUs were obtained. Dilution curves of intestinal microorganisms, culture-water microorganisms and mint root microorganisms for the three species of fish were basically flattened when the sequencing data were sampled and analyzed. This indicated that the number of species in this sample did not increase significantly with the number of sequences, and the amount of sequencing data was sufficient to reflect the majority of microbial diversity information in the sample (Fig. 1).

### Microbial species diversity analysis

**Fig. 2:**
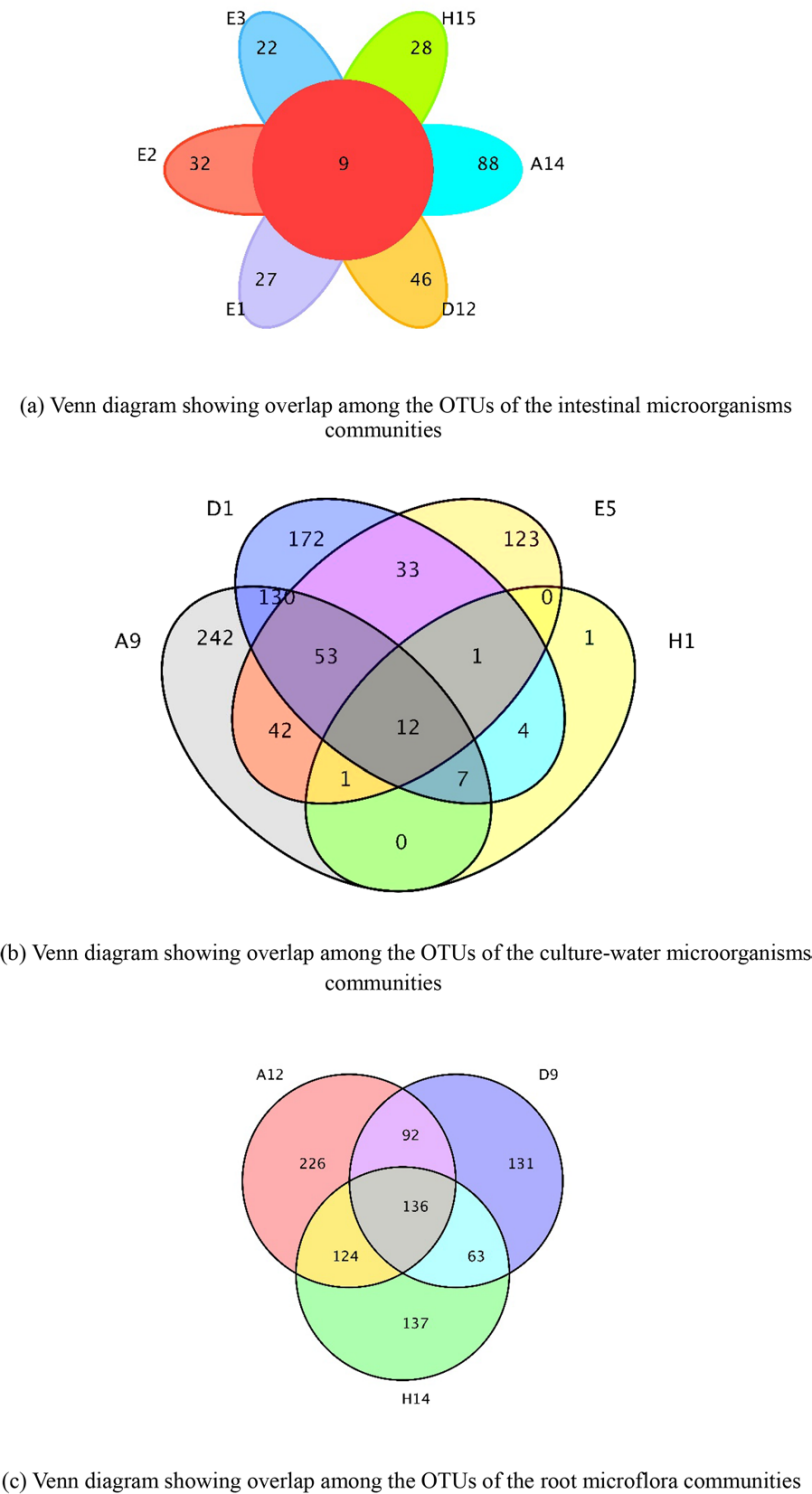
Venn diagram showing overlap among the OTUs of the intestinal microorganisms communities, culture-water microorganisms communities and root microflora communities

Differences in the numbers of specific and shared OTUs in different types of biological crusts are shown in Fig. 2. It can be seen that mint feeding had almost no effect on carp intestinal microbial OTUs (166 and 163), while crucian carp fed with mint had fewer intestinal microbial OTUs than the control group (122 and 163). Grass carp fed with mint had more intestinal microbial OTUs than grass carp in the conventional feeding group (199 and 137). The two groups of samples contained nine common arithmetic taxonomic units, accounting for 3.7% of the total OTUs of intestinal microorganisms. OTUs in carp culture water with mint were 83.8% more than the number in control water; grass carp culture water with mint had 55.5% more OTUs than the control, while the number of microbial OTUs in crucian carp culture water was 90.2% lower than that in the control. There were 12 shared OTUs in the four culture-water samples, accounting for 1% of the total OTUs in the culture water. Mint rhizosphere microbial OTUs showed that the highest number of mint rhizosphere microbial OTUs were found in carp culture buckets (578) while the lowest number of mint rhizosphere microbial OTUs were found in grass carp culture buckets (422). The number of arithmetic taxonomic units shared by the three groups of samples was 136, accounting for 9.32% of the total OTUs of the three groups of mint samples.

The α-diversity results showed a coverage of more than 92% for all species in the samples, and the sequencing depth covered all species in the samples (Table 2). The Ace and Chao1 indices of A14 and A9 were higher than those of E1 and E5, respectively, indicating that the intestinal bacterial community and the community richness of the culture-water community of carp were higher than those of the control groups after feeding mint. The Ace index and Chao1 index of H15 and H1 were lower than those of E2 and E5, respectively, indicating that the intestinal floral community and the community richness of culture water for crucian carp were reduced by feeding mint compared with the control group. The Ace index of D12 was lower than that of E3 and the Chao1 index was higher than that of E3, but the Ace index and Chao1 index of D1 were both much higher than those of E5, which indicated that the richness of the bacterial community in the culture water of grass carp was substantially higher than that of the control group, while the richness of the intestinal community of grass carp was slightly lower. A14, A9 and D1 had higher Shannon indices than their respective controls, and H15, D12 and H1 all had lower Shannon indices than their respective controls. Mint feeding increased the diversity of intestinal and culture-water communities of carp, decreased the diversity of intestinal and culture-water communities of crucian carp, and increased the diversity of the culture-water community of grass carp although the diversity of the intestinal community decreased. The Alpha diversity indexes of mint rhizosphere microorganisms showed that the Ace index, Chao1 index and Shannon index of A12 were higher than those of D9 and H14, indicating that the abundance of the mint rhizosphere microbial community was the highest and its diversity was the greatest in the carp culture bucket.

**Table 2:** Bacterial community richness of intestinal microorganisms, culture-water microorganisms and root microflora analyzed using the fish-flower (mint) symbiosis model

	Sample	OTU	Ace	Chao1	Shannon	Simpson	Coverage
Intestinal microorganisms	E1	166	215.9025	207.129	3.4428	0.6695	0.986
	A14	163	353.4197*	359.95*	5.627*	0.9164	0.962
	E2	163	205.6733	199.3846	5.2638	0.9414	0.988
	H15	122*	183.5986*	179.5*	3.3545*	0.766	0.988
	E3	137	295.5457	202.619	5.1096	0.9359	0.978
	D12	199*	264.2746*	247.4872*	4.8591	0.8553	0.979
Culture-water microorganisms	E5	265	472.7202	374.2	5.4732	0.9212	0.969
	A9	487*	714.2381*	727.0128*	7.7792*	0.9903	0.921
	H1	412*	55.6349*	35*	1.3072*	0.4307	0.999
	D1	26*	734.3969*	577.0698*	6.2927	0.9504	0.948
Root microflora	A12	578	787.385	767.3495	7.8949	0.9912	0.949
	D9	460	782.4826	642.6849	6.8528	0.9778	0.943
	H14	422	659.555	725.5167	7.1679	0.9721	0.942

^*^Significant difference compared with the control group; p < 0.05

**Fig. 3:**
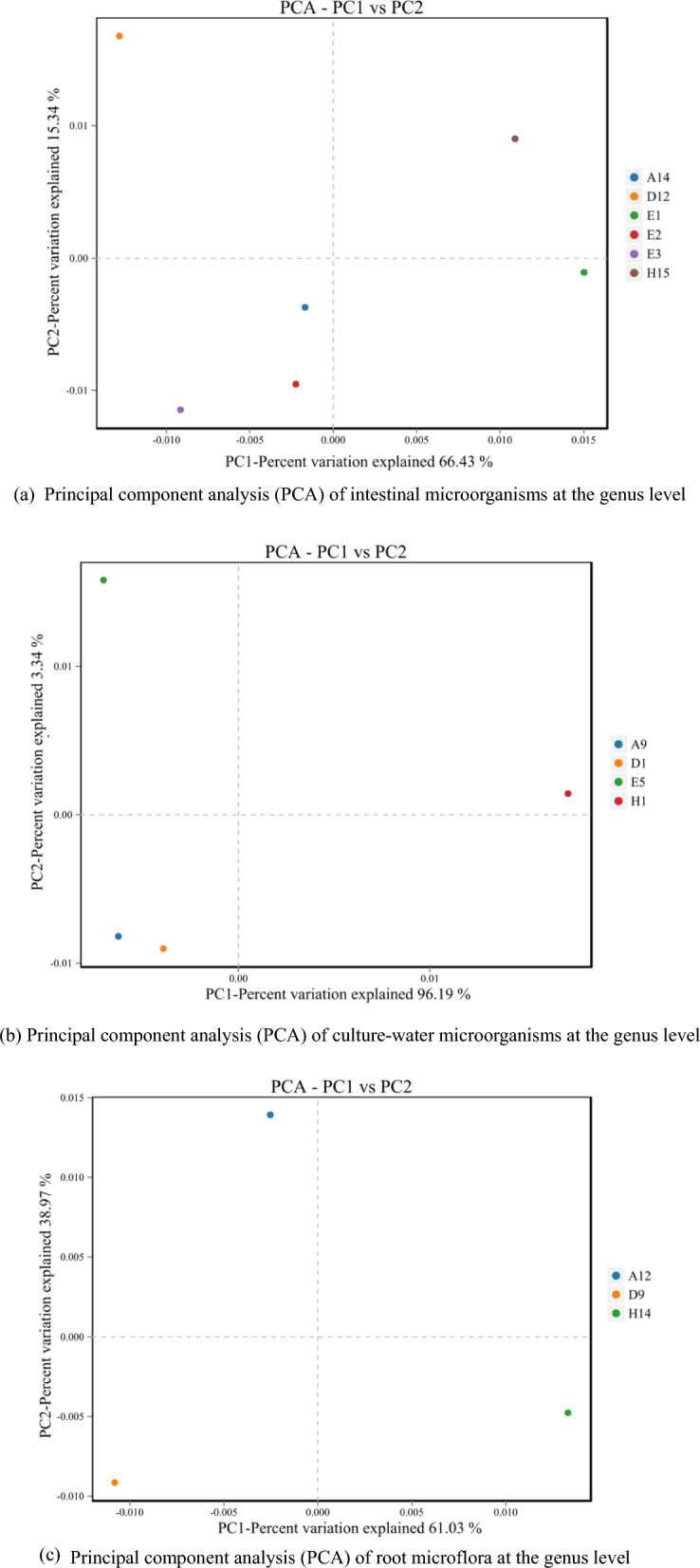
Principal component analysis (PCA) of intestinal microorganisms, culture-water microorganisms and root microflora at the genus level

Based on the OTU levels from the sequencing results, we performed β-diversity analysis for intestinal, water column and mint root microorganisms associated with each of the three species of fish using the PCA method. The contributions of PC1 and PC2 to differences in fish intestinal microorganisms were 66.43% and 15.34%, respectively, explaining 82.68% of the total variance, respectively (Fig. 3a). This indicates that the intestinal microorganisms of the three fish species were significantly altered and similarity in community composition was low. The cumulative contribution of PC1 and PC2 to the differences in microorganisms in culture water was 99.53% (Fig. 3b), and the culture-water samples for all three fish were distant from the control samples, indicating that the experimental and control culture-water bacterial communities were different. The PCA contribution of the three root samples is shown in Fig. 3c, with 61.03% for PC1 and 36.97% for PC2. It can be seen from the figure that the three root samples were very loosely distributed and far from each other, indicating that all three samples showed a degree of independence. The above data showed that the respective intestinal microorganisms of the three fish species fed with mint were significantly different from those of the control groups. At the same time, the microbial community of the cultured water body was also changed by the joint influence of different fish and mint, and the microorganisms attached to the mint root system were also significantly different.

### Microbial community composition analysis

**Fig. 4:**
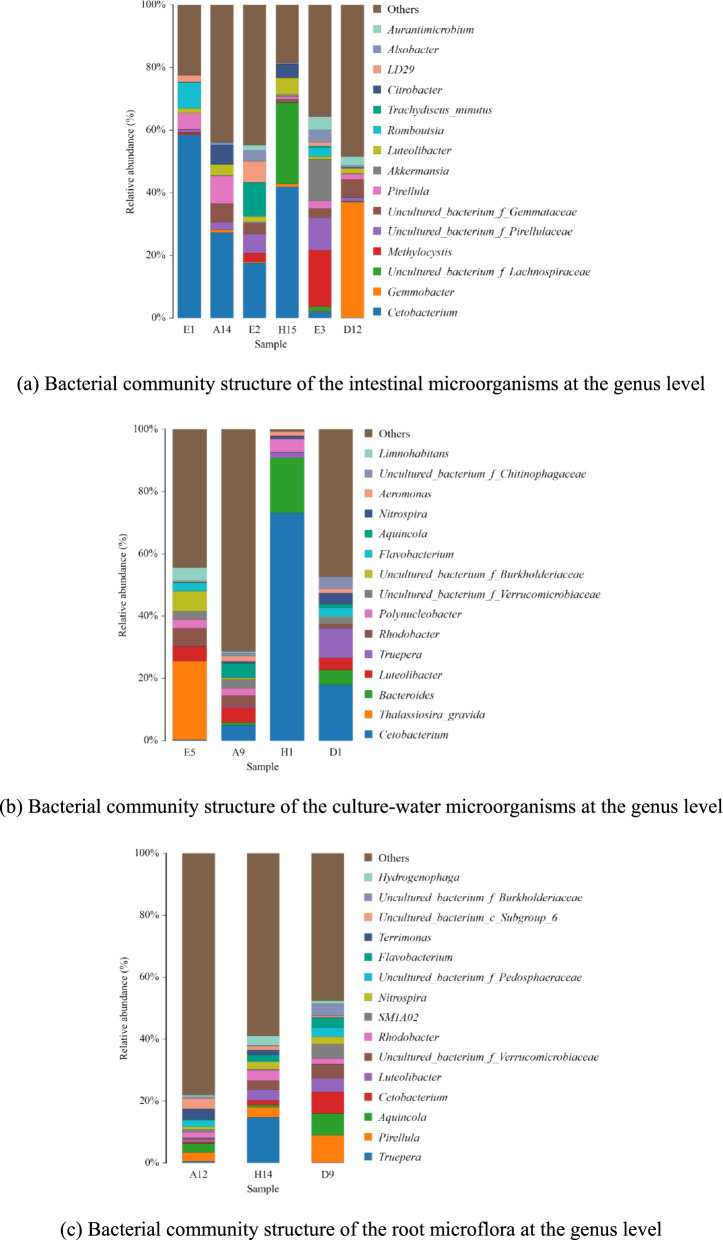
Bacterial community structure of the intestinal microorganisms, culture-water microorganisms and root microflora at the genus level

From the analysis of microbial community composition shown in Fig. 4a, it can be seen that the composition and abundance of the main bacteria in the intestinal microbial communities of the three fish species changed to different degrees after feeding with mint. At the genus level, *Cetobacterium* (58.58%), *Romboutsia* (8.23%) and *Pirellula* (5.17%) were predominant in the intestines of the carp control. In the experimental group of carp, the intestinal microorganisms were mainly *Cetobacterium* (27.31%), *Pirellula* (8.77%), *Citrobacter* (5.99%), *uncultured_bacterium_f_Gemmataceae* (5.83%) and *Luteolibacter* (3.52%). The original intestinal microbial community of carp changed obviously after mint feeding, with the abundance of *Cetobacterium* decreasing by 53.38% and that of *Citrobacter* increasing from 0.11% to 5.99%. *Cetobacterium* (17.66%), *Trachydiscus_minutus* (10.55%), LD29 (6.73%), *uncultured_bacterium_f_Pirellulaceae* (6.20%), *Alsobacter* (3.43%) and *uncultured_bacterium_f_Gemmataceae* (3.27%) were predominant in the intestines of the crucian carp control group. In the experimental group of crucian carp, intestinal microorganisms were mainly *Cetobacterium* (41.88%), *uncultured_bacterium_f_Lachnospiraceae* (25.84%), *Luteolibacter* (5.38%) and *Citrobacter* (4.38%). *Carassius auratus* (crucian carp) showed a significant increase in *Cetobacterium* in the intestine after feeding with mint. *Uncultured_bacterium_f_Lachnospiraceae* and *Citrobacter*, which were weakly represented in the intestine of the control carp, became the major genera of intestinal microorganisms in the experimental group, with 25.84% and 4.38%, respectively. The grass carp control intestine was dominated by *Methylocystis* (18.02%), *Akkermansia* (13.35%), *uncultured_bacterium_f_Pirellulaceae* (10.39%), *Aurantimicrobium* (4.29%) and *Alsobacter* (3.92%). In the grass carp experimental group, intestinal microorganisms were mainly *Gemmobacter* (36.97%), *uncultured_bacterium_f_Gemmataceae* (5.69%) and *Aurantimicrobium* (2.74%). The original dominant intestinal flora of grass carp was almost completely disrupted, with *Methylocystis*, which was the dominant genus, drastically reduced along with other originally dominant genera after feeding mint; meanwhile, *Gemmobacter*, which originally accounted for only 0.21%, increased in abundance to 36.97% after feeding mint.

The microbial community composition and abundance in the culture water of the three fish species differed significantly after feeding mint (Fig. 4b). At the genus level, the control water samples were dominated by *Thalassiosira_gravida* (25.26%), *uncultured_bacterium_f_Burkholderiaceae* (6.54%), *Rhodobacter* (5.76%), *Luteolibacter* (4.64%), *Limnohabitans* (4.19%) and *Polynucleobacter* (2.78%). In the experimental systems with mint, *Cetobacterium* (4.96%), *Luteolibacter* (4.92%), *Aquincola* (4.39%), *Rhodobacter* (3.52%) and *Polynucleobacter* (2.17%) were predominant in carp culture water, *Cetobacterium* (73.23%), *Bacteroides* (17.60%) and *Polynucleobacter* (4.21%) were predominant in crucian carp culture water, and *Cetobacterium* (18.02%), *Truepera* (9.54%), *Bacteroides* (4.71%), *Luteolibacter* (3.85%), *uncultured_bacterium_f_Chitinophagaceae* (3.84%), *Nitrospira* (3.63%) and *Flavobacterium* (2.95%) were predominant in grass carp culture water bodies. *Cetobacterium* was predominant in water bodies of all three groups of mint-fed cultured fish, especially in crucian carp culture water bodies, where *Cetobacterium* accounted for 73.23%, echoing the significant increase of *Cetobacterium* in the intestinal tracts of the experimental group of crucian carp. *Thalassiosira_gravida*, which was dominant in the control water, was significantly less abundant in all three experimental water bodies, leading us to speculate that some type of substance might be present in mint that would inhibit the growth of *Thalassiosira_gravida*.

The mint rhizosphere microorganisms in carp culture buckets were mainly *Terrimonas* (3.48%), *uncultured_bacterium_c_Subgroup_6* (3.24%), *Aquincola* (3.01%) and *Pirellula* (2.86%). The mint root microorganisms in the crucian carp culture bucket were mainly *Truepera* (14.73%), *Luteolibacter* (3.46%), *Rhodobacter* (3.30%), *Pirellula* (3.27%), *Hydrogenophaga* (3.17%) and *uncultured_ bacterium_f_Verrucomicrobiaceae* (2.95%). Mint root microorganisms in grass carp culture buckets were mainly *Pirellula* (8.83%), *Aquincola* (6.99%), *Cetobacterium* (6.99%), *uncultured_bacterium_f_Verrucomicrobiaceae* (4.59%), *SM1A02* (4.59%), *Luteolibacter* (4.40%), *uncultured_bacterium_f_Burkholderiaceae* (3.92%) and *Flavobacterium* (3.26%). This indicates that the root microflora of mint was substantially altered (Fig. 4c).

**Fig. 5:**
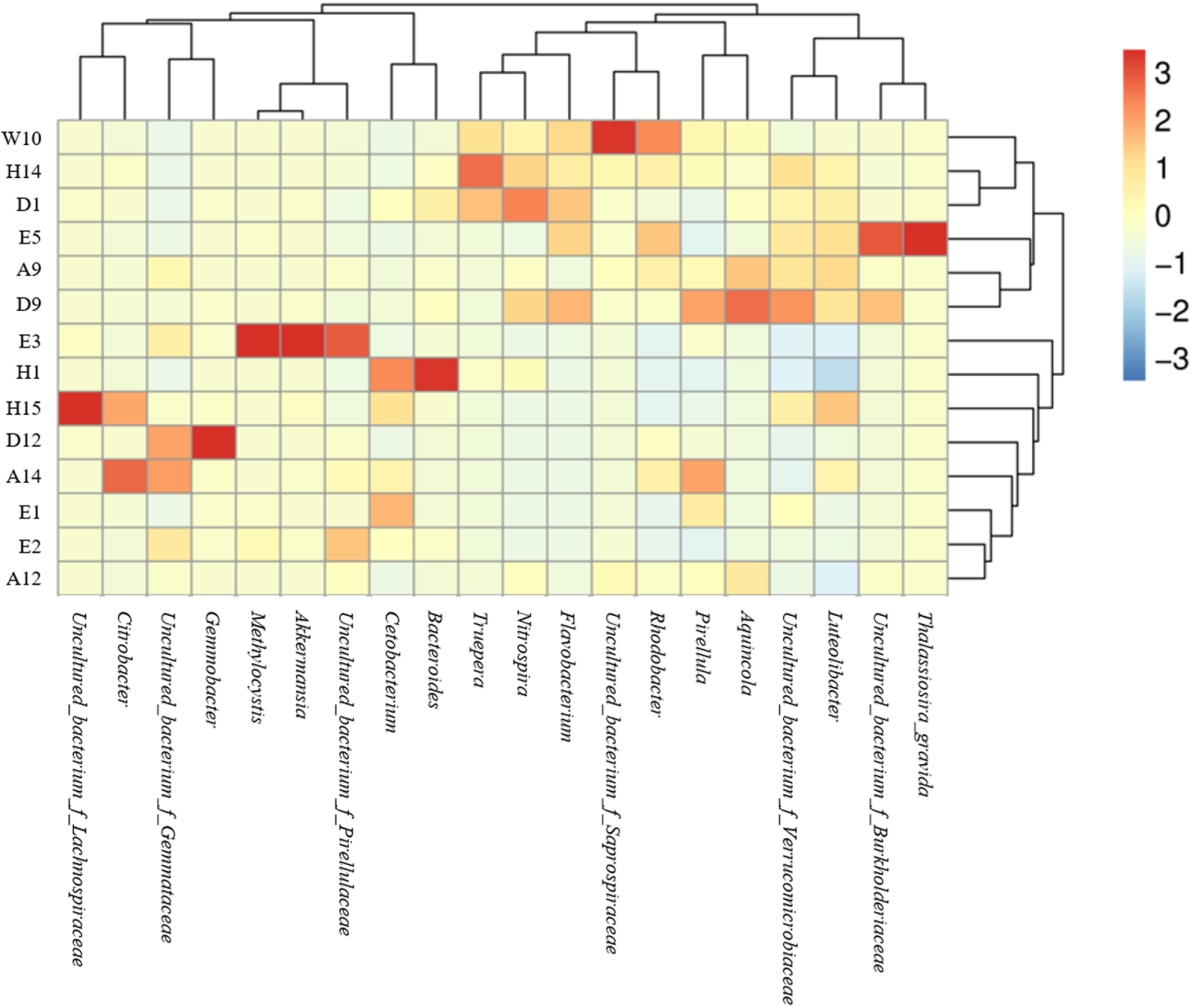
Heatmap of species abundance (the top 20 genera)

Heat map plots and sample clustering tree analysis of the effect of mint feeding on the composition of the microbial communities in fish intestines, culture water and mint roots at the genus levels for the three fish species are shown in Fig. 5. Overall, samples from each experimental group did not cluster significantly with the control groups. Fish intestinal microorganisms and culture-water microorganisms in the experimental group were significantly altered, and their microbial abundances were obviously different among the three groups of mint root samples. The main reason for this phenomenon was the excessive variation in the characteristic flora among samples. It is worth noting that samples of the same type did not cluster well among the three groups of intestinal microorganisms, aqueous microorganisms and root microorganisms. This further indicated that some of the genera spread widely among the three types of samples and caused some degree of influence on the original genera of the samples.

## Discussion

Studies have shown that in addition to culture water temperature, habitat and fish development rate, feed formulation also affects fish intestinal microbes (Carola et al. 2017; Bajinka et al. 2020). Chinese herbs have the effect of regulating substance metabolism, for example, *Coptis chinensis* significantly increases the levels of blood lysozyme and complement C3 and C4 in *Megalobrama amblycephala*, slowing down the process of oxidative stress and reducing the rate of apoptosis after feeding a high-fat diet (Chen et al. 2016). The addition of licorice to fish feed significantly enhances the immunity and promotes the growth of Nile tilapia (Abdel-Tawwab et al. 2021). However, the effect of mint addition on the intestinal bacteria of several common fish species is not clear. Peppermint contains several types of compounds such as volatile oils, flavonoids, organic acids, trace elements, triterpenes and sterols that are beneficial to fish growth (Chiou et al. 2020). This is why it is crucial to raise awareness of the effects of mint on fish growth.

In the present study, we found that *Cetobacterium* was dominant in the intestinal flora of both carp and crucian carp with or without mint feeding. This genus has been identified as a major component of freshwater fish microorganisms (Van Kessel et al. 2011). *Cetobacterium* isolated from fish intestines has been reported to produce Vitamin B_12_ (Castaneda-Monsalve et al. 2019). By contrast, Vitamin B_12_ is considered an essential nutrient for healthy growth and development of fish-like species and has the effect of promoting appetite and improving growth performance of fish (Hansen et al. 2013). It is important to note that the growth trends of *Cetobacterium* in the intestinal tract of the two fish species were different after feeding mint. *Cetobacterium* in the intestine of carp decreased by 53.38%, while *Cetobacterium* in the intestine of crucian carp increased significantly, suggesting that mint feeding was more helpful for promoting the growth of crucian carp. Although there was a decrease in *Cetobacterium* in the carp intestine, *Citrobacter* increased substantially, from 0.11% to 5.99%, and became the dominant genus of intestinal microorganisms in carp. *Citrobacter* in the intestine of crucian carp also increased by 4.38% after feeding mint. Studies have shown that *Citrobacter* is a genus of bacteria that facilitates the acquisition of large amounts of energy by fish from high-fat diets (Zhang et al. 2020). Energy conservation is essential for the survival of individual fish, and fat is a common form of energy reserve (Riddle et al. 2020). Therefore, we speculate that feeding mint to carp may help with fattening. *Romboutsia* or *Trachydiscus_minutus* was also present in large quantities among the intestinal microorganisms of carp and crucian carp in the control group of this study. This differed from previous studies showing that the second most dominant genera are *Aeromonas*, *Bacteroides* or *Citrobacter*, respectively (Li et al. 2018; Meng et al. 2021). This may be due to geographical differences of the test sampling sites. We also found that the intestinal microorganisms of grass carp in the control group were dominated by *Methylocystis* and *Akkermansia*, which is slightly different from the findings of Lu et al. (2020) and Huang et al. (2020). *Methylocystis* is a Type II aerobic methane-oxidizing bacterium with an important role in methane abatement and carbon cycling (Rumah et al. 2021). *Akkermansia* is a heavy metal tolerant bacterium and has the ability to utilize nitrite (Xu et al. 2022). Both genera are beneficial bacteria that maintain intestinal health (Guo et al. 2022; Ma et al. 2023). In the mint-fed group, the original dominant intestinal flora of grass carp was almost completely disrupted, and *Methylocystis*, which was the predominant genus, was significantly reduced along with other dominant genera, while the abundance of *Gemmobacter*, which was only 0.21% under control culture conditions, increased to 36.97% after feeding mint. *Gemmobacter* is widely present in lake sediments (Qu et al. 2020); however, its presence in large quantities in fish has not been reported. This genus is one that has a remarkable ability to utilize methylamines (Kröber et al. 2021). Methylamines and nitrogenous heterocyclic substances such as indoles, thiazoles and amides are the main contributors to fishy odors in aquatic animals (Kawaguchi et al. 2019). We speculate that a significant decrease in *Methylocystis* and *Akkermansia* after feeding mint was the main reason for an increase in methylamines in fish. The significant increase of *Gemmobacter* after feeding mint facilitated the degradation of methylamines, effectively reducing the fishy odors of grass carp. Changes in the structure of the intestinal bacterial community reflect the overall health status of the host animal (Xiong et al. 2015). In the present study, mint feeding produced representative changes in the intestinal microorganisms of each of the three fish species, which were overall beneficial to the growth and development of the three fish species.

*Cetobacterium* was not the dominant group in the control water; however, the dominant genus in all three fish culture water bodies in the experimental group was *Cetobacterium*. The relative abundance of *Cetobacterium* in crucian carp culture water was as high as 73.23%, and this result was consistent with the significant increase in the relative abundance of *Cetobacterium* in the intestinal tract of crucian carp. It is noteworthy that *Thalassiosira gravida*, which was dominant in the control water, was completely absent in all three experimental groups. *Thalassiosira gravida* is a native microalga with low temperature and high radiation tolerance (Lacour et al. 2018). Studies have shown that menthol has an inhibitory effect on the growth of *Microcystis aeruginosa* (Hu et al. 2014). Menthol is the main substance in peppermint essential oil (Chanotiya et al. 2021). An inhibitory effect of mint on *Thalassiosira gravida* has not been reported. We speculated that some type of substance may be present in mint that inhibited the growth of *Thalassiosira gravida*. Overall, the addition of mint resulted in a significant increase in the number of microbial genera suitable for fish growth in the water column and inhibited the growth and reproduction of algae in the culture water, which contributed to improvement of the conditions of the culture water.

The microbial composition of the mint root systems differed in each of the three fish culture barrels. *Terrimonas* are a class of denitrifying microorganisms that play an important role in reducing nitrate (NO^3−^) and lowering nitrite (NO^2−^) levels in the water column (). Analysis of the water quality of the carp culture showed that the nitrite content was slightly lower than that in the other two groups, indirectly indicating that *Terrimonas* is beneficial for the improvement of water quality in cultured water bodies. *Truepera* was the dominant genus in the mint rhizosphere of the crucian carp group. Studies have shown *Truepera* to be effective in reducing antibiotic resistance by reducing and stopping the spread of antibiotic resistance genes (Li et al. 2021). The consumption of peppermint roots by crucian carp may be helpful in reducing its drug resistance. *Pirellula* is a typical anaerobic ammonia oxidizing bacterium that can use the NO^3−^-N decomposition product NO^2−^-N to oxidize NH^4+^-N to produce N_2_ under low oxygen conditions for denitrification (Luna et al. 2022). Analysis of water quality also revealed that the overall ammonia nitrogen content of grass carp culture water was better than that of the water associated with the other two fish species. Mint has a good ability to purify water bodies (Xu et al. 2019); on the one hand, mint absorbs nutrients from water bodies during its growth, and on the other hand, mint rhizosphere microorganisms may uptake and degrade nutrient salts.

*Pirellula* found in the rhizosphere was also widespread in the intestines of the three fish species, and the genera *Truepera*, *Aquincola*, *Rhodobacter*, *Flavobacterium* and *uncultured_bacterium_f_Burkholderiaceae* were also found in culture water. *Cetobacterium* and *Luteolibacter* were found in fish intestines, culture water and the mint rhizosphere. Therefore, we speculate that the fish intestine, culture water and mint root systems in the experimental groups maintained their own specific genera while other genera were extensively exchanged between the three.

In this study, we fed three fish species mint in a fish-flower (mint) symbiosis system and investigated the microbial community structures of fish intestinal microorganisms, culture-water microorganisms and mint root microorganisms with the help of high-throughput sequencing technology and bioinformatics analysis. We revealed the beneficial effects of mint feeding on the structure and diversity of microbial communities of fish, water bodies and mint roots in the fish-flower symbiosis model, providing a theoretical basis for the promotion and application of fish-flower (mint) symbiosis technology and healthy fish culture technology. The literature exploring the effects of mint on fish intestinal microorganisms and water microorganisms in fish-flower symbiosis models is limited, and although some progress has been made in this study, more research on fish quality and fish intestinal bacteria cultured in different regions is needed to further promote the progress of the culture technology and improve fish quality.

## Data Availability

All data generated or analysed during this study are included in this published article (and its supplementary information files).
